# Mandibular osteomyelitis induced by denosumab administration: Case report

**DOI:** 10.1097/MD.0000000000047148

**Published:** 2026-01-16

**Authors:** Chongyuan Liu, Lidi Cheng, Ting Jiang, Jian Fang, Ling Zhang

**Affiliations:** a Huzhou Central Hospital, Fifth School of Clinical Medicine of Zhejiang Chinese Medical University, Huzhou, Zhejiang Province, China; b Huzhou Central Hospital, Affiliated Central Hospital of Huzhou University, Huzhou, Zhejiang Province, China; c Department of Stomatology, Anji County Second People’s Hospital, Huzhou, Zhejiang Province, China; d Department of Stomatology, Anji County BTown Central Health Center, Huzhou, Zhejiang Province, China.

**Keywords:** denosumab, mandibular osteomyelitis, osteoporosis

## Abstract

**Rationale:**

Denosumab is a commonly used agent for the management of osteoporosis, particularly in elderly populations. The adverse reaction associated with its use is osteomyelitis of the jaw, which poses significant clinical challenges, especially in patients with compromised overall health.

**Patient concerns:**

The patient was an elderly individual with osteoporosis and a documented history of denosumab treatment. At the time of consultation, the primary manifestations included pain, swelling, and purulent discharge at the extraction socket, which impaired normal eating and quality of life. The patient urgently sought safe and effective treatment to alleviate discomfort and restore oral function.

**Diagnoses:**

Based on the patient’s clinical manifestations, history of denosumab treatment, imaging studies, and laboratory tests, the final diagnosis was confirmed as “denosumab-induced mandibular osteomyelitis.”

**Interventions:**

For the elderly patient with compromised overall health, an individualized comprehensive regimen combining conservative treatment and surgical debridement under local anesthesia was adopted.

**Outcomes:**

Following the comprehensive intervention combining conservative treatment and surgical debridement under local anesthesia, the patient’s clinical manifestations improved significantly: the surgical wound healed well.

**Lessons:**

This case emphasizes that clinicians must maintain heightened vigilance and consider alternative treatment options for vulnerable populations receiving denosumab therapy. Additionally, it highlights the urgent need for further research to thoroughly elucidate the pathogenic mechanisms and risk factors underlying denosumab-induced mandibular osteomyelitis.

## 1. Introduction

Medication-related osteomyelitis of the jaw (MRONJ) is gaining increasing attention in clinical practice, with oral health professionals predominantly linking it to bisphosphonates or certain tumor-targeted drugs.^[1]^ The existing literature indicates that an incidence of jaw necrosis with intravenous administration of traditional anti-osteoporotic bisphosphonates is approximately 0.2%.^[2,3]^ With the approval and introduction of a novel anti-osteoporotic agent, denosumab, into the Chinese market in June 2020 (EU approval in 2010), clinicians, particularly those in orthopedics, endocrinology, and related specialties, along with patients, are showing preference for denosumab over traditional bisphosphonates due to its milder gastrointestinal reactions and minimal impact on renal function.^[4]^ This paper aims to offer an overview of the evolving landscape of MRONJ, shedding light on the emergence of denosumab as a promising alternative to conventional bisphosphonate therapy.

Denosumab is currently the most potent receptor activator of nuclear factor-κB ligand (RANKL) inhibitor available for therapeutic use in humans. With high affinity for RANKL, it disrupts the interaction between RANKL and receptor activator of nuclear factor-κB (RANK), inhibiting the generation and function of osteoclasts.^[5,6]^ This mechanism leads to a reduction in bone resorption, an increase in bone mass, and an improvement in bone strength. However, denosumab is associated with the side effect of osteomyelitis of the jaw, a complication that clinicians may not be sufficiently aware of. Given the escalating market share of denosumab, this serious adverse event requires heightened attention from clinicians.^[7]^

Upon reviewing recent literature, it becomes evident that there is a scarcity of reported cases of osteomyelitis of the jaw associated with denosumab.^[8,9]^ Consequently, this report presents a case of osteomyelitis of the jaw in an elderly female patient with osteoporosis who developed this condition after 3 months of denosumab treatment. Significantly, the patient experienced a successful recovery following treatment in our department. This case report serves the dual purpose of augmenting clinicians’ awareness of denosumab-related osteomyelitis of the jaw and providing a valuable reference for successful treatment strategies.

## 2. Case report

**Figure 1.**
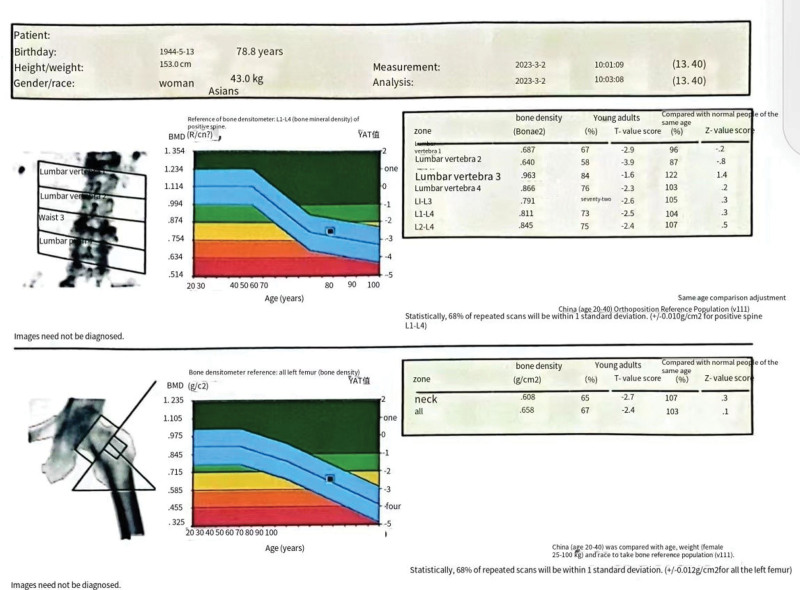
Bone density examination results.

The patient, a 79-year-old female, presented at our Department of Oral and Maxillofacial Surgery due to left mandibular swelling and pain, accompanied by gingival purulence, persisting for 2 months. In December 2021, the patient received a diagnosis of osteoporosis (Fig. 1) and initiated treatment with alendronate (bisphosphonate). In November 2022, the treatment was transitioned to subcutaneous denosumab administration (60 mg/6 months), supplemented with vitamin D and calcium. Approximately one month after initiating denosumab in December 2022, the patient reported pain in the anterior mandibular region. Subsequently, in March 2023, due to mobility and discomfort in the residual root of the lower left anterior tooth, the patient underwent extraction at an external facility. Iodine-soaked gauze was used to pack the socket, but subsequent persistent purulent discharge and ongoing pain promoted the patient to seek further medical attention. The patient has a medical history of hypertension, coronary heart disease, and a longstanding diagnosis of dry syndrome with associated pulmonary interstitial fibrosis. Long-term oral prednisolone has been a consistent component of the patient’s medical regimen.

**Figure 2.**
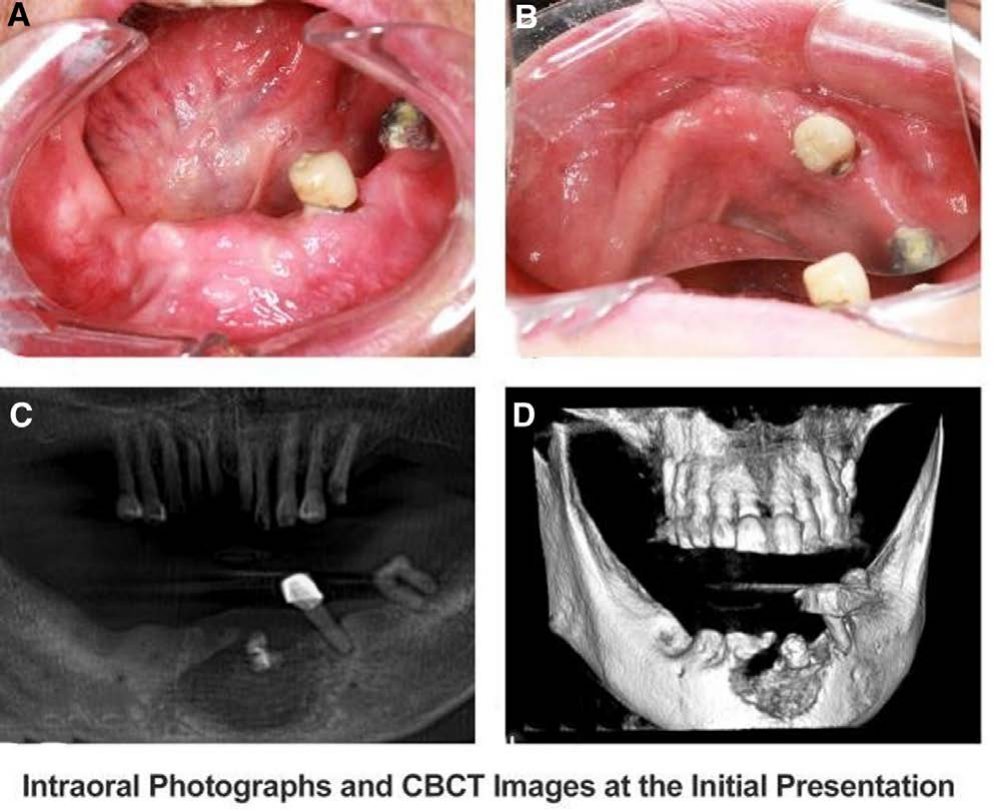
Photographs and CBCT images at the initial presentation. (A, B) Intraoral photographs showing the presence of a labial gingival fistula in the anterior mandibular region accompanied by abundant purulent discharge. (C) CBCT images revealed irregular, patchy low-density shadows in the anterior mandibular region, with peripheral areas displaying patchy areas indicative of necrotic bone. (D) Three-dimensional reconstruction showing cortical bone destruction with communication observed on both the labial and lingual aspects of the mandible. CBCT = cone-beam computed tomography.

Upon specialized examination, notable findings included swelling of the lower lip and chin, slightly erythematous skin, a two-finger mouth opening, gingival swelling in the 35 to 43 region, and the presence of a labial gingival fistula in the anterior mandibular region accompanied by abundant purulent discharge (Fig. 2A and B). Tooth 35 exhibited Grade II mobility. Intraoral cone-beam computed tomography (CBCT) imaging revealed irregular, patchy low-density shadows in the anterior mandibular region, with peripheral areas displaying patchy areas indicative of necrotic bone (Fig. 2C). Additionally, imaging revealed cortical bone destruction with communication observed on both the labial and lingual aspects of the mandible (Fig. 2D). The clinical diagnosis was stage II MRONJ.^[10]^

Considering the patient’s numerous underlying health conditions and the heightened surgical risks, coupled with the patient’s refusal of surgical intervention, a conservative treatment approach was adopted. The patient underwent a 5-day course of intravenous antibiotics for anti-inflammatory purposes (ceftriaxone sodium 2.0 grams once daily; metronidazole sodium chloride injection 0.5 grams once daily). Locally, alternating rinses with 0.9% saline solution and 1% hydrogen peroxide were administered. Simultaneously, family members were instructed to perform at-home rinsing of the intraoral fistula twice daily using a solution consisting of 0.9% saline and 1% hydrogen peroxide.

Two months later, the patient presented purulent discharge from the labial gingival fistula in the anterior mandibular region, with exposed block-shaped necrotic bone. CBCT imaging revealed extensive irregular high-density shadows with uneven density in the anterior mandibular region, indicative of block-shaped necrotic bone formation. With the patient’s informed consent, non-surgical debridement of the exposed necrotic bone was performed under local anesthesia to reduce irritation to the surrounding soft tissues. The region underwent repeated irrigation with 0.9% saline solution and 1% hydrogen peroxide. The patient was instructed to persist with at-home rinsing and dressing changes (following the established protocol) and scheduled for a follow-up visit 3 months later.

**Figure 3.**
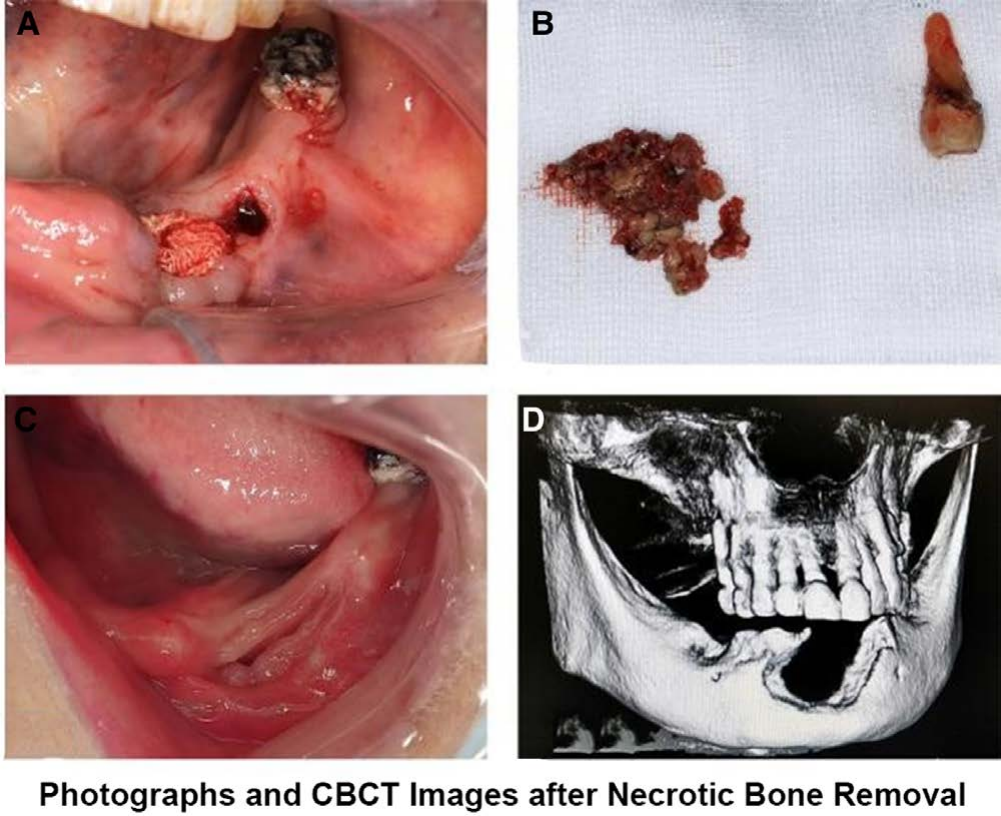
Photographs and CBCT images after necrotic bone removal. (A) Intraoral photographs showing the surgical site packed with iodine-soaked gauze. (B) Digital photographs displayed the excised necrotic bone and the extracted tooth. (C) Intraoral images demonstrated satisfactory surgical wound healing at one month postoperatively, characterized by the absence of evident redness or purulent discharge. (D) Postoperative CBCT imaging showing the absence of necrotic bone in the anterior mandibular region. CBCT = cone-beam computed tomography.

At the 3 months follow-up appointment, the patient continued to exhibit a small amount of purulent discharge from the labial gingival fistula in the anterior mandibular region, with complete exposure of necrotic bone and grade III mobility in tooth 35. Following the patient’s informed consent, complete removal of the exposed necrotic bone and extraction of tooth 35 were performed under local anesthesia. The surgical site was thoroughly irrigated with 0.9% saline solution, packed with iodine-soaked gauze (Fig. 3A). Postoperatively, the patient received a 3-day course of intravenous antibiotics for anti-inflammatory purposes (ceftriaxone sodium 2.0 grams once daily; metronidazole sodium chloride injection 0.5 grams once daily) to prevent infection. The patient was instructed to change the iodine-soaked gauze weekly. Figure 3B displays the excised necrotic bone and the extracted tooth.

One month after the removal of necrotic bone, the wound in the anterior mandibular region had significantly healed, characterized by the absence of pain, evident redness, or purulent discharge. The labial gingiva displayed enhanced resilience and growth in Figure 3C. CBCT imaging confirmed the absence of necrotic bone in the anterior mandibular region (Fig. 3D).

## 3. Discussion

MRONJ refers to the continuous exposure of facial bones lasting ≥ 8 weeks in patients undergoing treatment with anti-resorptive or anti-angiogenic drugs, without a history of head and neck radiation exposure. The maxilla is affected in 2/3 of cases, the mandible in 1/4, and involvement of both upper and lower jaws can occur simultaneously.^[10–12]^ Denosumab-induced jaw necrosis is exceedingly rare, but its incidence increases with prolonged therapy. The reported rates are 0.04% at 3 years, 0.06% at 5 years, and 0.66% at 10 years. In the extension phase of the phase III FREEDOM study, involving 4550 postmenopausal women receiving low-dose denosumab (60 mg/6 months) for 10 years to treat osteoporosis, 13 cases of jaw osteomyelitis were reported.^[13,14]^

Denosumab exerts its therapeutic effects by binding with high affinity to RANKL, thereby inhibiting the interaction between RANKL and RANK, and subsequently suppressing the generation and function of osteoclasts.^[15,16]^ It is plausible that denosumab may affect the normal remodeling and healing of the jawbone.

The first reported case of denosumab-related osteomyelitis of the jaw dates back to 2010, and subsequent similar case reports have since emerged. Patients who develop jaw osteomyelitis in relation to denosumab exposure often have a history of risk factors, including tumors, smoking, diabetes, corticosteroid use, periodontal disease, early contact points of dentures, and notably, invasive oral procedures (e.g., tooth extractions).^[11,17,18]^ In the present case, the patient exhibited multiple predisposing factors – including diabetes, long-term corticosteroid therapy, and chronic periodontitis – indicating a multifactorial contribution to MRONJ development.

Because the patient was initially treated at an external institution, early radiological data were unavailable. The CBCT image presented in this report was obtained approximately 5 months after denosumab therapy began. The CBCT revealed an extensive bone defect, suggesting that necrosis might have developed earlier, potentially during the prior alendronic acid treatment period, due to cumulative suppression of bone turnover.

In this case, MRONJ was most likely associated with denosumab therapy, given the clear temporal relationship between the initiation of denosumab and the onset of jaw pain, as well as the occurrence of tooth extraction during the period of active denosumab effect. However, we acknowledge that the patient’s prior exposure to oral alendronate may have contributed to a cumulative inhibition of bone remodeling, thereby increasing susceptibility to osteonecrosis.^[19]^ Several studies have suggested that sequential or combined use of bisphosphonates and denosumab may potentiate MRONJ risk due to prolonged antiresorptive activity within the bone.^[20,21]^ Therefore, while denosumab was considered the primary precipitating factor, the potential additive effect of prior bisphosphonate therapy cannot be completely excluded.

During the course of treatment, the patient’s serum calcium and phosphorus levels remained within normal ranges. However, the alkaline phosphatase (ALP) level gradually declined – from 100 U/L in March 2022 to 40 U/L in September 2023 (reference range: 50–135 U/L) – indicating a sustained reduction in bone metabolic activity. Because the patient’s osteoporosis management was initially conducted in the rheumatology department of another hospital, laboratory data such as PTH, vitamin D, and bone resorption markers were not available and were self-reported by the patient. Clinically and radiographically, extensive bone necrosis was observed, and only necrotic bone tissue was identifiable within the defect area, suggesting that the actual extent of necrosis might have been underestimated radiographically.

Before initiating denosumab therapy, it is essential to evaluate systemic and local risk factors. Thorough communication and dental examination can enhance patients’ awareness of oral health and allow for the management of potential complications. Dentists should exercise caution when performing invasive procedures (e.g., extractions, implants) or treating conditions such as ill-fitting dentures, severe caries, periodontal disease, or intraoral infections in patients under denosumab therapy. When invasive dental procedures are unavoidable, clinicians should make individualized risk–benefit assessments and consult with the attending physician regarding possible drug discontinuation or substitution. Monitoring bone turnover markers may also provide valuable guidance for dental treatment timing.

In this case, no adjunctive therapies such as hyperbaric oxygen or platelet-rich fibrin were applied. Considering the patient’s advanced age, multiple comorbidities, and refusal of extensive surgical intervention, a conservative approach was chosen – including local irrigation, antibiotic therapy, and non-surgical debridement.^[11]^ This regimen achieved gradual healing without the need for additional modalities, demonstrating that individualized conservative therapy can still yield satisfactory outcomes in medically complex patients.

According to the American Association of Oral and Maxillofacial Surgeons guidelines, conservative therapy (mouth rinses and antibiotics) is recommended for stages 0 and 1 MRONJ, while combined conservative and surgical interventions are advised for stages 2 and 3.^[22]^ In the present case, given the patient’s age and systemic comorbidities, conservative management combined with local debridement under anesthesia resulted in favorable healing, aligning with American Association of Oral and Maxillofacial Surgeons recommendations.

## 4. Conclusion

MRONJ is a rare but severe complication, and the widespread use of denosumab has given rise to complications related to jaw osteomyelitis. This case underscores the necessity for close attention to oral health in patients undergoing denosumab treatment for osteoporosis, with a focus on promptly identifying and addressing potential MRONJ complications. Therefore, clinicians should conduct comprehensive oral examinations before initiating denosumab therapy, strive to mitigate risk factors during treatment, and regularly monitor the health of the jawbone to minimize the occurrence of MRONJ. It is crucial to recognize that this report serves as a reference and does not establish a direct causal relationship between denosumab and jaw osteomyelitis. Further investigation into the potential link between denosumab and MRONJ requires additional clinical research and data.

## Author contributions

**Conceptualization:** Ling Zhang.

**Data curation:** Ting Jiang, Jian Fang.

**Investigation:** Lidi Cheng.

**Project administration:** Ling Zhang.

**Visualization:** Chongyuan Liu.

**Writing – original draft:** Chongyuan Liu, Lidi Cheng, Ting Jiang, Jian Fang.

**Writing—review & editing:** Chongyuan Liu, Lidi Cheng, Ling Zhang.
